# IN.PACT AV Access randomized trial update: 3-year clinical results by anatomic and demographic subsets and 5-year extension mortality analysis

**DOI:** 10.1186/s42155-026-00746-z

**Published:** 2026-08-19

**Authors:** Andrew Holden, Hiroaki Haruguchi, Kotaro Suemitsu, Naoko Isogai, Jeffrey Hull, Bret N. Wiechmann, Hong Wang, Jennifer Seamans, Robert Lookstein

**Affiliations:** 1 Department of Interventional Radiology, Auckland Hospital, Auckland, New Zealand; 2 Haruguchi Vascular Access Clinic, Tokyo, Japan; 3 Osaka Keisatsu Hospital, Osaka, Japan; 4 Shonan Kamakura General Hospital, Kanagawa, Japan; 5 Richmond Vascular Center, Richmond, VA USA; 6 Vascular and Interventional Physicians, Gainesville, FL USA; 7 Peripheral Vascular Health, Medtronic, Plymouth, MN USA; 8 Department of Diagnostic, Molecular, and Interventional Radiology at the Icahn School of Medicine at Mount Sinai, New York City, NY USA

**Keywords:** Drug-coated balloon, PTA, Arteriovenous fistulas, Radiocephalic, Brachiocephalic, Brachiobasilic, Peri-anastomotic, Cephalic arch, Venous outflow, Race, Sex

## Abstract

**Purpose:**

To report 36-month outcomes by anatomic and demographic subsets, and mortality through 60 months from the IN.PACT AV Access Study, a prospective, multicenter, randomized trial of a paclitaxel drug-coated balloon (DCB) versus percutaneous transluminal angioplasty (PTA) for dysfunctional hemodialysis arteriovenous fistulas (AVF).

**Methods:**

The IN.PACT AV Access Study enrolled 330 participants at 29 international sites and randomized 1:1 to DCB (*n* = 170) or PTA (*n* = 160). Target lesion primary patency (TLPP) outcomes through 36 months were stratified by core laboratory-adjudicated anatomic subsets: lesion types (de novo and restenotic), AVF type (radiocephalic and brachiocephalic/ brachiobasilic), and lesion locations (peri-anastomotic, cephalic arch, and venous outflow). TLPP outcomes were also stratified by participant demographics. Mortality rates were reported through 60 months.

**Results:**

Through 36 months, DCB showed numerically or statistically greater TLPP compared to PTA in de novo (50.6% versus 42.2%, *p* = 0.175) and restenotic (40.5% versus 22.7%, *p* < 0.001) lesions. Similarly, 36-month TLPP was significantly better for DCB compared to PTA in radiocephalic (44.5% versus 33.8%, *p* = 0.039) and brachiocephalic/ brachiobasilic (39.9% versus 21.3%, *p* = 0.011) AVFs. In lesion location subsets, 36-month TLPP was numerically greater with DCB versus PTA: peri-anastomotic (40.4% versus 31.1%, *p* = 0.047), cephalic arch (40.9% versus 27.9%, *p* = 0.079), and venous outflow (45.6% versus 25.5%, *p* = 0.048). Through 36 months, TLPP favored DCB treatment over PTA across race and sex. After incorporating additional vital status information, sixty-month freedom from all-cause mortality was 59.0% DCB versus 53.5% PTA (*p* = 0.510).

**Conclusion:**

Results showed sustained TLPP benefit for DCB compared to PTA in all subset analyses through 36 months and no mortality signal for DCB through 5 years.

**Trial registration:**

ClinicalTrials.gov NCT03041467. Registered 01 February 2017.

**Level of evidence:**

Level 1b, Randomized controlled trial.

**Graphical Abstract:**

**Figure:**
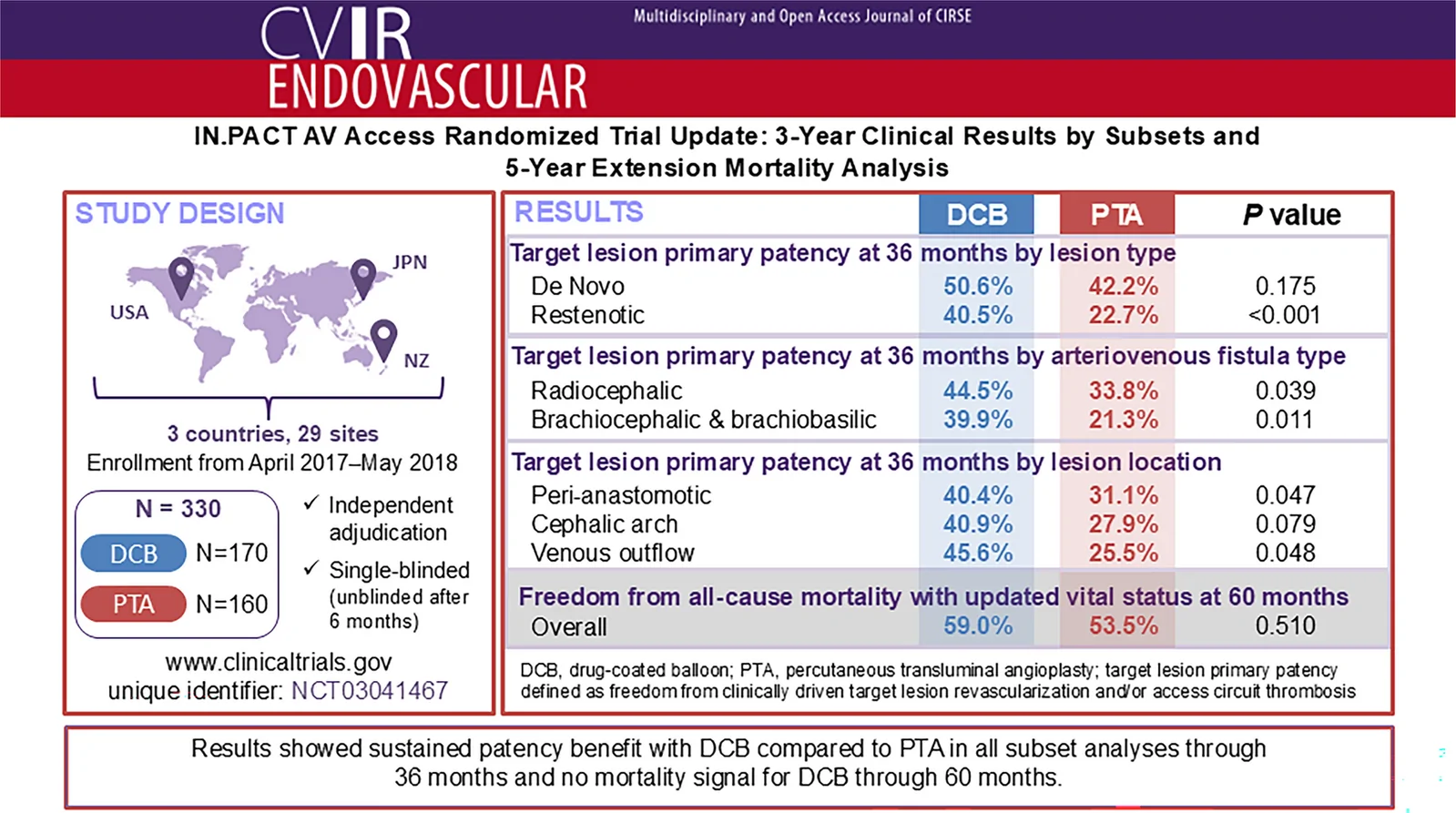

**Supplementary Information:**

The online version contains supplementary material available at 10.1186/s42155-026-00746-z.

## Introduction

Chronic kidney disease is a highly prevalent global condition, affecting about 10% of the general population worldwide [1, 2]. In the United States alone, an estimated 37 million adults have kidney disease [3], of whom more than 808,000 are living with end-stage kidney disease (ESKD) and require ongoing dialysis or a kidney transplant [4]. For ESKD patients who need permanent vascular access in order to receive long-term hemodialysis, an autogenous arteriovenous fistula (AVF) is recommended as the preferred initial access, according to guidelines from the National Kidney Foundation’s Kidney Disease Outcomes Quality Initiative (KDOQI) and other international guidelines [5–7].

Treatment of dysfunctional AVFs is an important aspect of ESKD patient care as hemodynamically significant stenoses and thrombotic events are common in dialysis access circuits [8]. Although a variety of endovascular techniques are used as first-line treatments for dysfunctional AVFs, drug-coated balloons (DCBs) have emerged as a promising option [9–11]. The IN.PACT AV Access Study was the first investigational device exemption DCB trial that met the designed primary endpoints [12] and demonstrated long-term safety and effectiveness through 36 months [13, 14]. However, there is limited information on whether the added benefits of DCBs are sustained across demographics, AVF subtypes, or lesion characteristics.

The objective of this post-hoc analysis is to assess the outcomes of a DCB compared to standard percutaneous transluminal angioplasty (PTA) in various subsets within the IN.PACT AV Access Study through 36 months. Another aim of this analysis is to report long-term mortality rates in the IN.PACT AV Access Study using the 5-year follow-up data.

## Methods

### Trial design

The IN.PACT AV Access Study (www.ClinicalTrials.gov; NCT03041467) was a prospective, global, multicenter, single-blinded, 1:1 randomized controlled trial (RCT) to assess the efficacy and safety of the IN.PACT AV DCB versus PTA for the treatment of patients presenting with de novo or restenotic lesions of a native AVF in the upper extremity. Participants were enrolled across 29 sites in the United States, Japan, and New Zealand between April 2017 and May 2018 (Supplementary Table S1). The details of the trial design have been reported previously [12–14]. The study was conducted in accordance with good clinical practice guidelines and the Declaration of Helsinki. The ethics committee or institutional review board of each site reviewed and approved the trial protocol and amendments. All the participants provided written informed consent prior to any study-specific procedures. The study oversight included an independent data monitoring board (NAMSA, formerly known as Syntactx, New York, New York, USA), an independent Clinical Events Committee (CEC) responsible for the adjudication of adverse events and clinical endpoints (Syntactx), independent core laboratories to analyze duplex ultrasonography images (VasCore, Massachusetts General Hospital, Boston, Massachusetts) and angiographic images (Syntactx). Data were analyzed by Medtronic and the Baim Institute for Clinical Research (formerly the Harvard Clinical Research Institute; Boston, MA, USA).

### Randomization and index procedure

Patients who provided informed consent and met all inclusion/exclusion criteria [12–14] were randomized following successful predilation with a high-pressure PTA balloon to reach a residual stenosis ≤ 30% of the lesion based on the operator’s clinical judgement. No other devices, such as cutting/scoring balloons or DCBs, were allowed for predilation. Randomization was achieved by an interactive voice/web/mobile-based system set up through a third-party vendor (Bracket, now Signant Health; San Francisco, California). Participants were randomized 1:1 to either IN.PACT AV DCB (Medtronic) or PTA. For the DCB, the balloon length needed to exceed the target lesion by approximately 1 cm at either side to ensure full coverage. If the total target lesion length was longer than the longest available balloon, an additional DCB was used with overlap of approximately 1 cm. A second dilatation was required with a non-DCB if the initial dilation resulted in residual stenosis > 30% by visual estimate or the presence of flow-limiting dissection.

### Follow-up

This trial was originally designed to follow participants for 24 months; however, the trial was extended to follow participants through 36 months for clinical outcomes and through 60 months for mortality at the recommendation of the United States Food and Drug Administration (FDA). Follow-up assessments were scheduled for 30 days and 3, 6, 9, 12, 18, 24, and 36 months post-index procedure. Vital status (alive or not alive) was collected through 60 months post-index procedure. Efforts were also made to collect vital status on any participants who exited the study in the United States for reasons other than death. For the participants in Japan and New Zealand, once a participant exited the study, no medical information could be retrieved.

### Analysis endpoints

The present analysis reports target lesion primary patency (TLPP) after DCB compared to PTA stratified by lesion type, AVF type, lesion location, race and sex through 36 months. TLPP was defined as freedom from clinically driven target lesion revascularization or access circuit thrombosis. Definitions of lesion locations are shown in Supplementary Fig. S1. An event was adjudicated as a clinically driven target lesion revascularization if the target lesion had a ≥ 50% diameter stenosis (per angiographic core laboratory assessment) in the presence of clinical or physiological abnormalities that indicated dialysis access dysfunction, or a ≥ 70% stenosis without the presence of clinical or physiological abnormalities indicating dialysis access dysfunction. Additionally, freedom from all-cause mortality was assessed through 60 months.

### Statistics

All analyses were conducted based on participants’ randomization assignments and included those with evaluable data. In addition to reporting mortality outcomes through 60 months, post-hoc analyses were stratified by these subgroups: target lesion type (de novo and restenotic), AVF type (radiocephalic and brachiocephalic/brachiobasilic), target lesion location (peri-anastomotic, cephalic arch, and venous outflow), sex (male and female), and race (Asian, Black, and White).

Time-to-event outcomes were evaluated using the Kaplan–Meier methods, and event-free probabilities were estimated over time for each treatment group. Participants were censored at withdrawal, loss to follow-up, AVF abandonment, or death, as applicable. Two-sided 95% confidence intervals (CIs) for time-to-event outcomes in each treatment group were computed using a log–log transformation. Differences between Kaplan–Meier curves were assessed with the log-rank test, and hazard ratios (HRs) with two-sided 95% CIs were calculated. Treatment-by-subgroup interaction tests were performed to assess potential heterogeneity of treatment effect. Annual cutoffs for statistical analysis were set at 360 days per year (e.g., 1,080 days for the 3-year cutoff). No adjustment was made for multiple comparisons, and a two-sided significance level of 0.05 was used. Statistical analyses were performed using SAS version 9.4 (SAS Institute, Cary, NC) and R version 4.5.1 (R Foundation for Statistical Computing, Vienna, Austria) [15].

## Results

### Participants

**Fig. 1:**
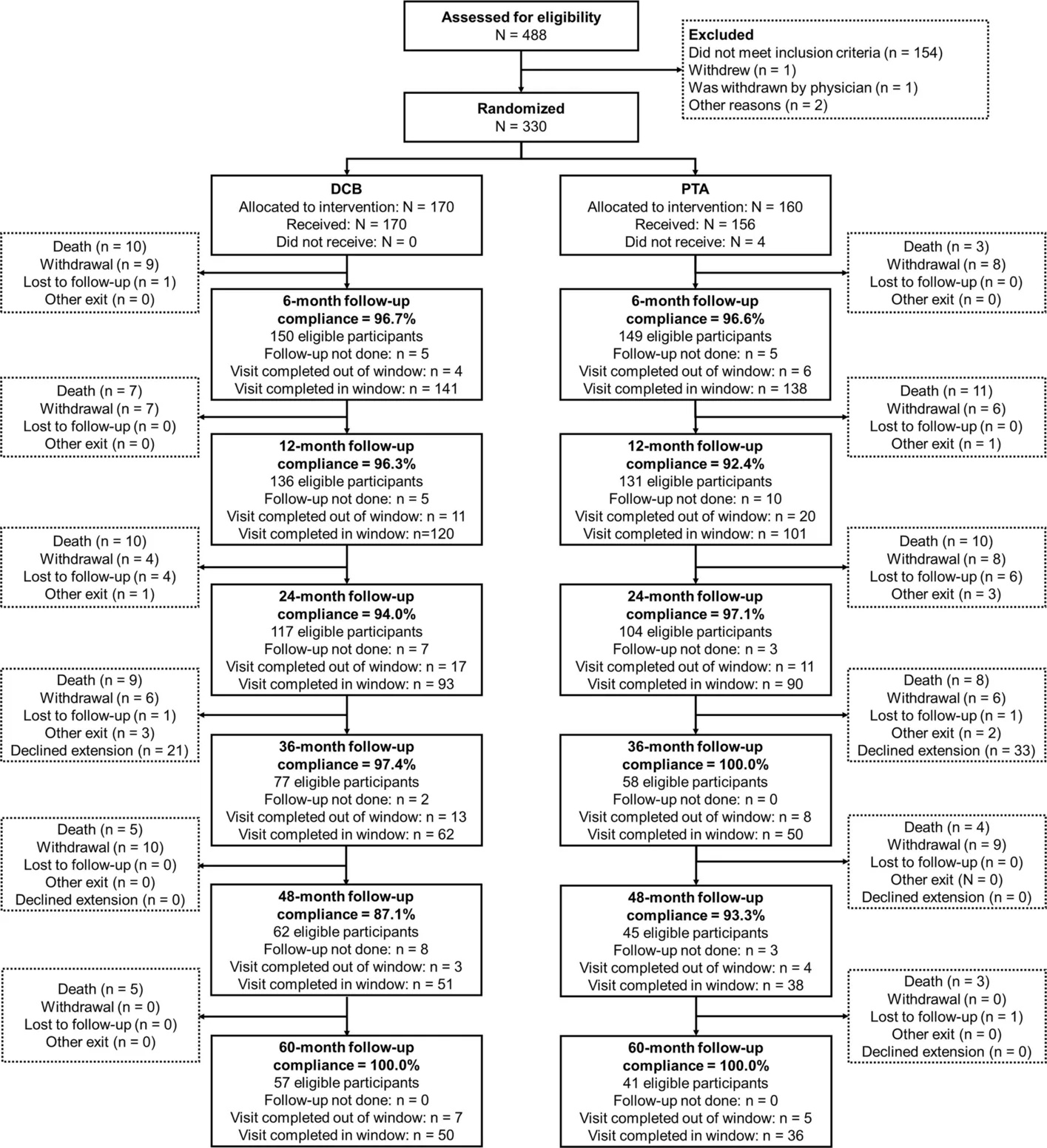
CONSORT diagram showing participant flow through 60 months in the IN.PACT AV Access Study. DCB, drug-coated balloon; PTA, percutaneous transluminal angioplasty

The participant flow is shown in a CONSORT diagram (Fig. 1). A total of 330 participants were randomized across 29 global sites, with 170 assigned to the DCB group and 160 to the PTA group. The follow-up compliance remained high through 60 months in both groups. Baseline characteristics were reported previously [13].

### Target lesion primary patency by subsets through 36 months

**Fig. 2:**
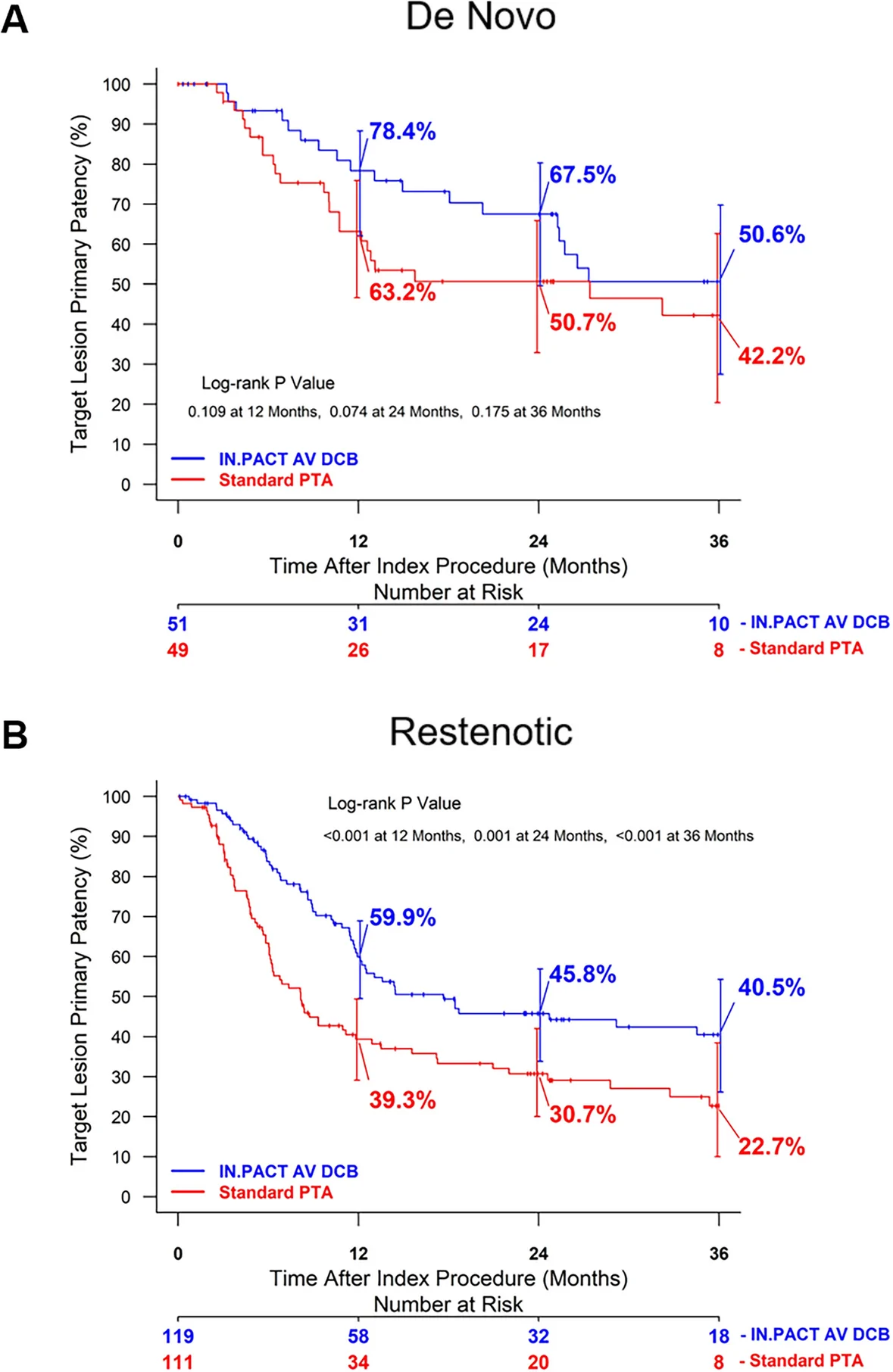
Target lesion primary patency by lesion type. Kaplan–Meier curves of target lesion primary patency in de novo lesions (**A**) and restenotic lesions (**B**). Bars represent 95% confidence intervals. DCB, drug-coated balloon; PTA, percutaneous transluminal angioplasty

Subset analysis by lesion type showed numerically higher TLPP for DCB compared to PTA for de novo lesions (50.6% versus 42.2%, *p* = 0.175; Fig. 2A) and significantly higher TLPP after DCB compared to PTA in restenotic lesions (40.5% versus 22.7%, p < 0.001; Fig. 2B) at 36 months.

**Fig. 3:**
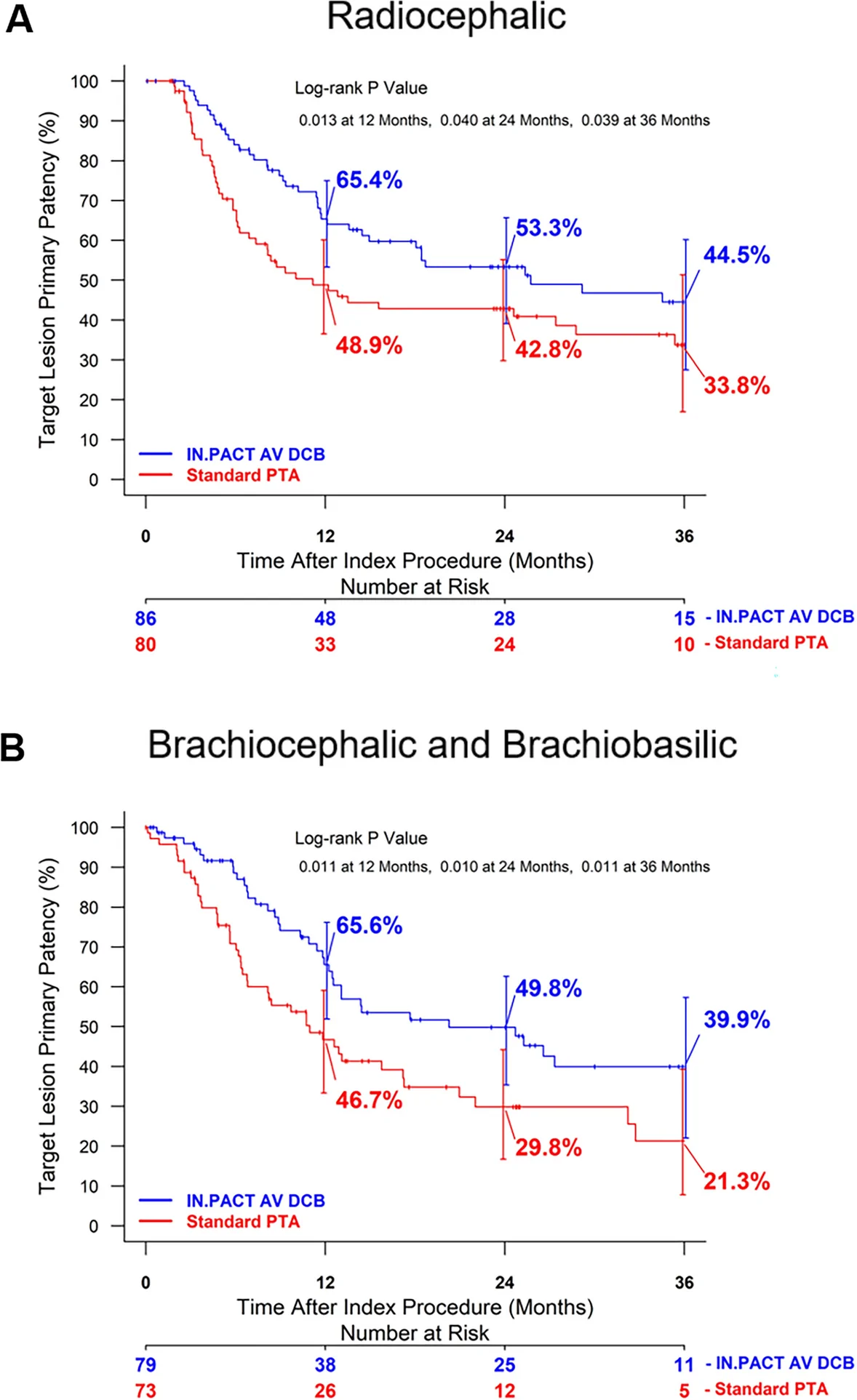
Target lesion primary patency by AVF type. Kaplan–Meier curves of target lesion primary patency in radiocephalic AVF (**A**) and brachiocephalic/brachiobasilic AVF (**B**). Bars represent 95% confidence intervals. AVF, arteriovenous fistula; DCB, drug-coated balloon; PTA, percutaneous transluminal angioplasty

In the subset analysis by AVF type, DCB demonstrated superior TLPP compared to PTA at 36 months whether it was forearm (radiocephalic) (44.5% versus 33.8%, *p* = 0.039; Fig. 3A) or upper arm (brachiocephalic/brachiobasilic) (39.9% versus 21.3%, *p* = 0.011; Fig. 3B).

**Fig. 4:**
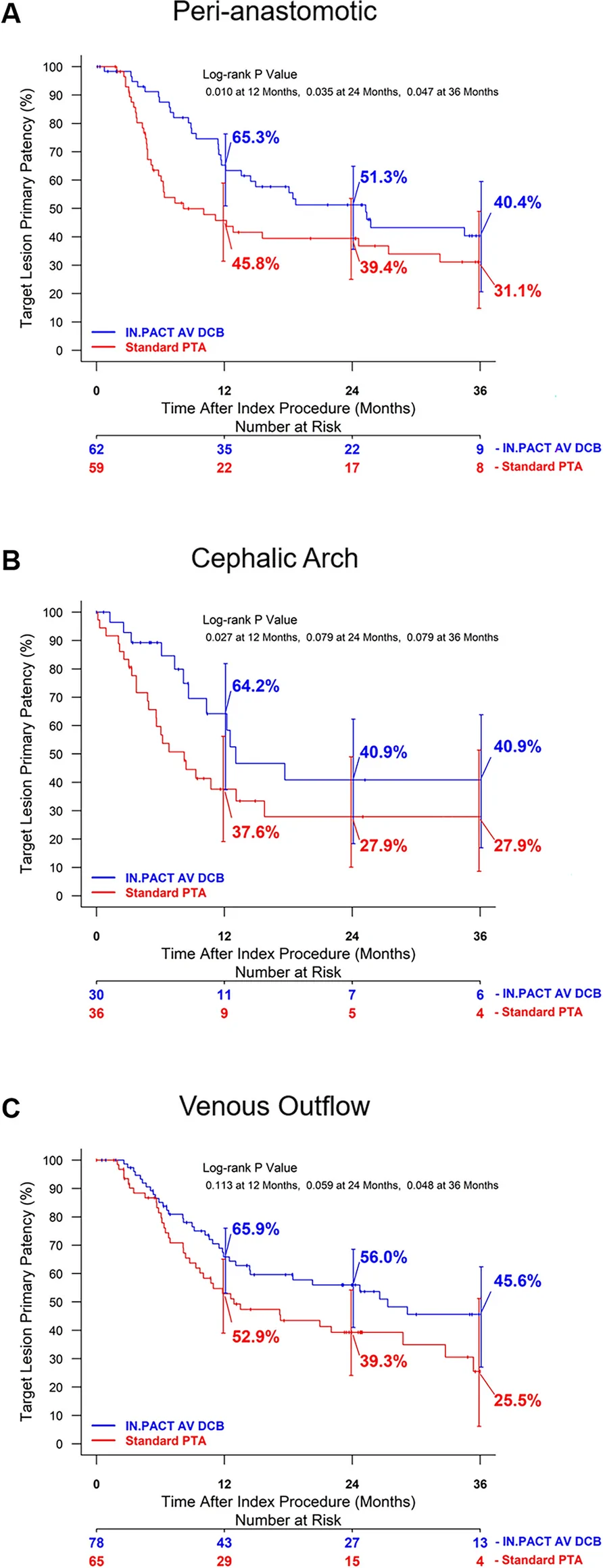
Target lesion primary patency by lesion location. Kaplan–Meier curves of target lesion primary patency based on lesion location: peri-anastomotic (**A**), cephalic arch (**B**), and venous outflow (**C**). Bars represent 95% confidence intervals. DCB, drug-coated balloon; PTA, percutaneous transluminal angioplasty

There was a trend toward higher TLPP with DCB compared to PTA when analyzed by lesion location (Fig. 4), with a statistically significant benefit for DCB in peri-anastomotic (40.4% versus 31.1%, *p* = 0.047; Fig. 4A) and venous outflow locations (45.6% versus 25.5%, *p* = 0.048; Fig. 4C) at 36 months. In cephalic arch locations, TLPP favored DCB over PTA with a statistically significant difference at 12 months (64.2% versus 37.6%, *p* = 0.027) but not at 36 months (40.9% versus 27.9%, *p* = 0.079; Fig. 4B).

### Subset analyses by race and sex through 36 months

**Fig. 5:**
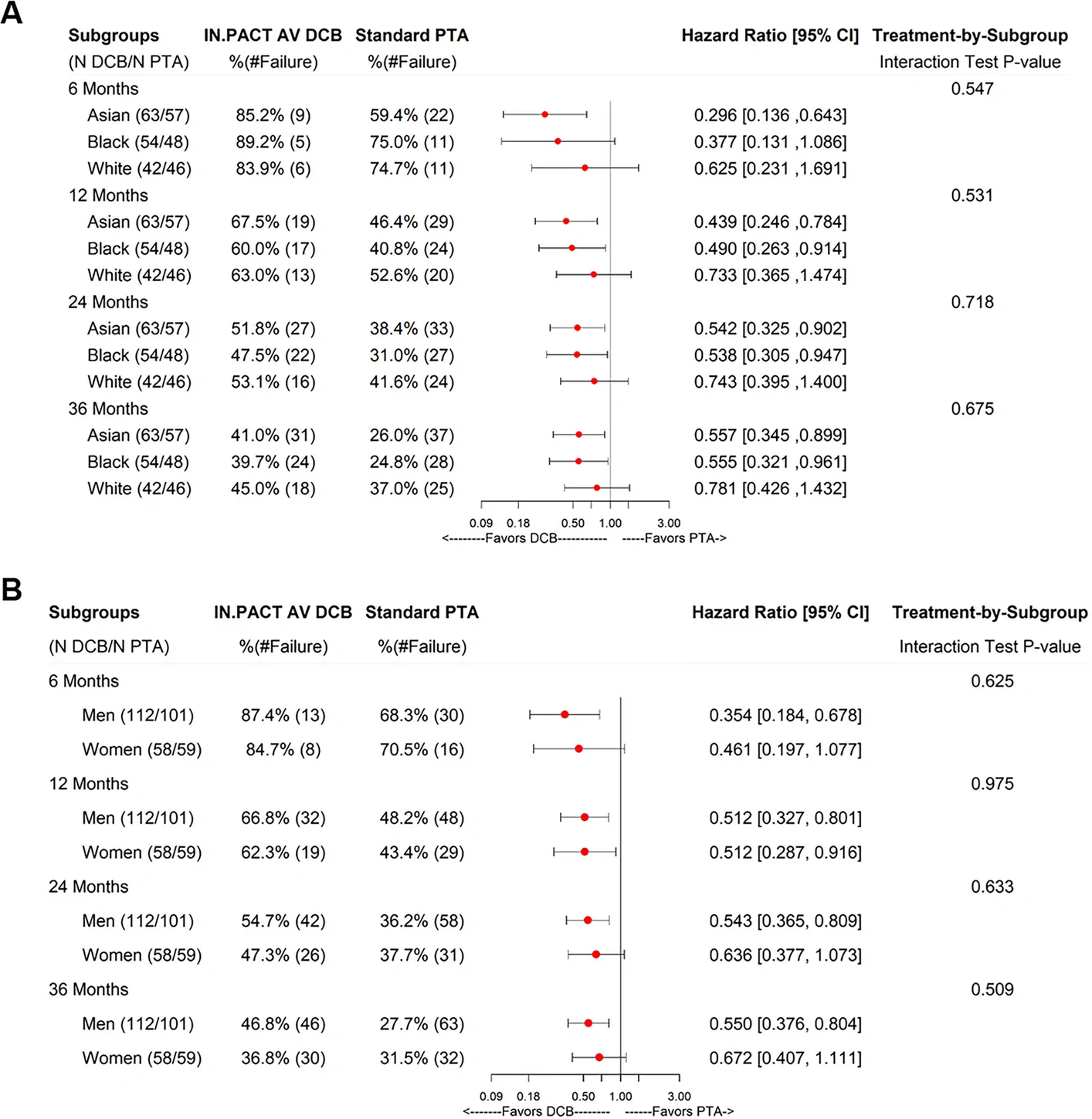
Target lesion primary patency by demography. Forest plot of Kaplan–Meier estimated TLPP rates by race (**A**) and by sex (**B**) with hazard ratio (95% CI). CI, confidence interval; DCB, drug-coated balloon; PTA, percutaneous transluminal angioplasty

Subset analyses by race and sex are presented in Fig. 5. Through 36 months, TLPP favored DCB treatment over PTA in all races included in the analysis (Asian, Black and White); however, no statistically significant treatment effect by race interactions were observed (Fig. 5A). Similarly, DCB was favored over PTA for TLPP in both men and women through 36 months, though no statistically significant interactions were observed (Fig. 5B).

### All-cause mortality through 60 months

**Fig. 6:**
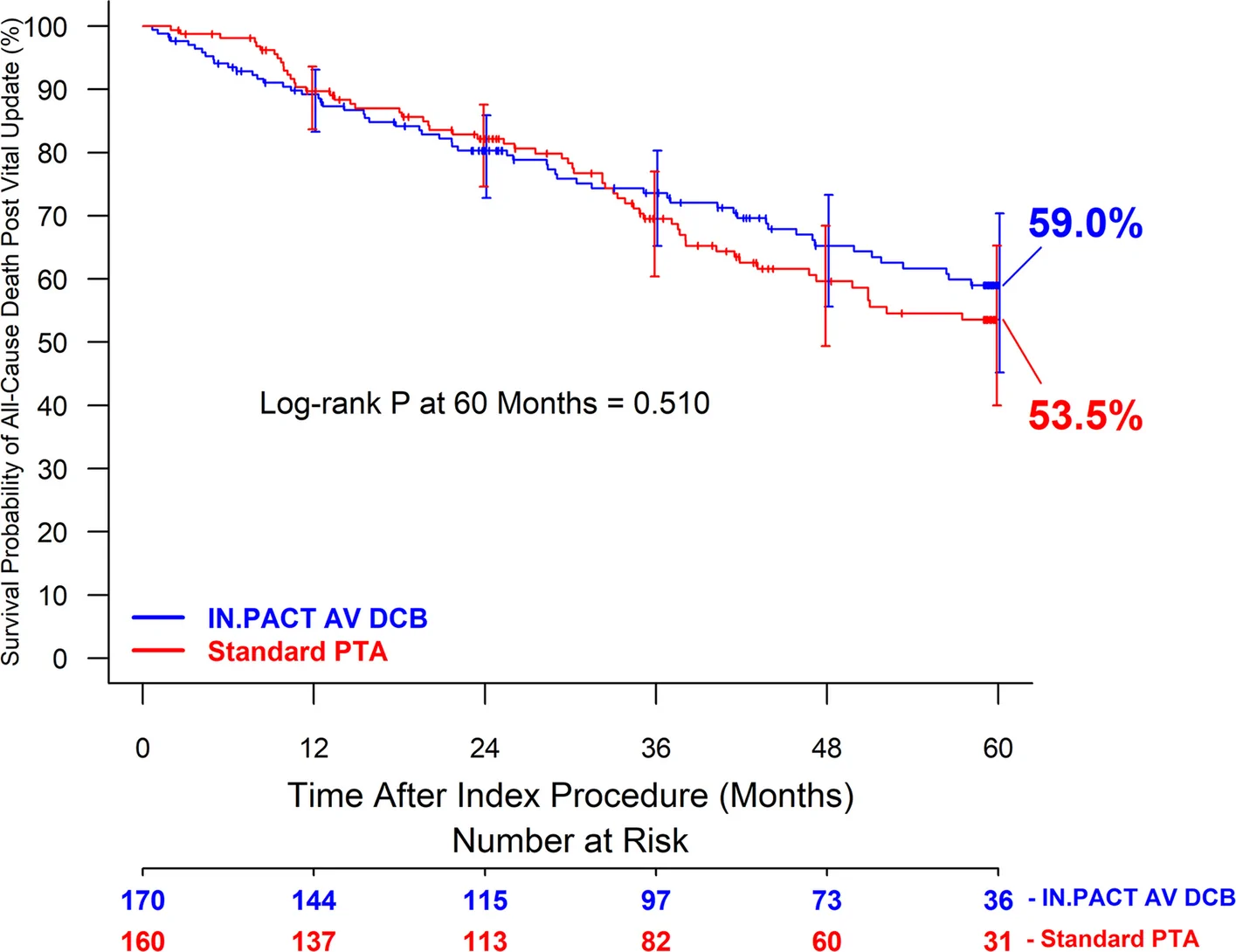
Freedom from all-cause mortality through 60 months. Kaplan–Meier curves of freedom from all-cause mortality with the updated vital status in the IN.PACT AV DCB and standard PTA groups through 60 months. Bars represent 95% confidence intervals. DCB, drug-coated balloon; PTA, percutaneous transluminal angioplasty

Long-term longitudinal assessment of survival outcome was conducted using the 60-month extension follow-up data. With vital status included, overall survival information was available in 75.9% (129) of participants in the DCB group and 73.8% (118) of participants in the PTA group through 60 months. At the 1800-day cutoff for analysis, 58 had died in the DCB group, and 60 had died in the PTA group. Freedom from all-cause mortality through 60 months did not differ significantly between DCB and PTA (59.0% versus 53.5%, *p* = 0.510; Fig. 6).

## Discussion

In this post-hoc analysis from the IN.PACT AV Access Study, DCB consistently outperformed PTA in sustaining TLPP across multiple subgroups and lesion types through 36 months. Notably, a more profound improvement in TLPP with DCB compared to PTA was observed in lesions known to be associated with more aggressive neointimal hyperplasia and those that are difficult to treat, such as restenotic, peri-anastomotic, and venous outflow lesions. DCB demonstrated superior benefits compared to PTA in both smaller forearm (radiocephalic) and larger upper arm (brachiocephalic/brachiobasilic) AVF conduits. While the advantage of DCB over PTA was consistent across races and sexes, no statistically significant interactions were observed. Importantly, there was no significant difference in long-term mortality between the DCB and PTA groups through 5 years of vital status follow-up, suggesting a lack of mortality signal with paclitaxel-coated balloons in this study.

To our knowledge, there are no other published long‑term (up to 3 years) prospective RCTs comparing DCBs versus PTA for AVF stenoses with stratification by anatomic and demographic subgroups. However, Trerotola et al. reported outcomes through 24 months from the multicenter Lutonix AV RCT comparing a different DCB (Lutonix DCB) to PTA in various subsets in the treatment of AVF stenosis [16]. This study found no significant difference between DCB and PTA in any of the subsets at 24 months [16]. A previous publication also reported that the Lutonix AV RCT did not meet the primary study endpoint at 6 months with TLPP of 71% for the Lutonix DCB and 63% for the PTA control (*p* = 0.06) [17]. The investigator-led multicenter PAVE RCT of the same DCB also failed to demonstrate a difference between DCB and PTA in terms of patency outcomes [18]. Some possible reasons for the differences in outcomes between the current IN.PACT AV Access RCT and the Lutonix RCTs may be related to device characteristics. The IN.PACT AV DCB is coated with a higher dose of paclitaxel (3.5 μg/mm^2^) using a urea-based excipient, whereas the Lutonix DCB uses a lower paclitaxel dose (2 μg/mm^2^) with a polysorbate and sorbitol-based excipient. Although the impact of these differences on clinical pharmacokinetics remains speculative, preclinical pharmacokinetic studies have shown sustained release and long-term tissue presence of paclitaxel with the IN.PACT DCB, while the Lutonix DCB demonstrated rapid early dissolution [19], which may explain the higher effectiveness of the urea-based IN.PACT DCB.

Neointimal hyperplasia, resulting from vascular injury and altered hemodynamics, is widely considered a common cause of AVF stenosis, whether the lesion is de novo or restenotic [20]. Restenotic lesions, however, are associated with more aggressive neointimal hyperplasia and poorer outcomes compared to de novo stenoses [21, 22]. In the present study, TLPP after PTA and DCB was lower in restenotic lesions than in de novo lesions through 36 months; however, TLPP was better sustained in the DCB group compared to PTA in restenotic lesions.

AVF stenosis most commonly occurs at sites of turbulent blood flow or cannulation, such as the peri-anastomotic location and in the venous outflow [23, 24]. In the current analysis, DCB showed significantly better TLPP than PTA at 36 months in peri-anastomotic (40.4% versus 31.1%) and venous outflow (45.6% versus 25.5%) lesions. Few studies reported 3-year outcomes in these subsets [25–27]. The PTA patency rates in the present study aligned with findings of earlier PTA studies [26, 27]; however, none reported on DCB.

Another important finding of this subset analysis was the sustained higher TLPP of DCB over PTA for cephalic arch stenosis treatment (40.9% versus 27.9% at 36 months). The cephalic arch is another common location for vascular access stenosis in dysfunctional hemodialysis AVF because of its unique anatomic characteristics [28]. Both endovascular and surgical approaches have been explored for managing cephalic arch stenosis, but there remains no clear consensus on which strategy is most effective because the underlying etiology of cephalic arch stenosis remains poorly understood [28]. Primary patency was reported as low as 23% at 12 months [29] and 17% at 36 months [30] after PTA, even with bailout stent placement. Cutting balloons did not improve patency rates in one retrospective study [31]. Bare metal stents are also limited by high in-stent restenosis [28] and low primary patency rates (< 30% at 1 year) [32], while stent grafts showed primary patency of 23.5% at 36 months in cephalic arch stenosis [33], a lower rate than the PTA group of the current IN.PACT AV Access Study. A few studies have evaluated the treatment effect of DCBs in the treatment of cephalic arch stenosis specifically, but both studies reported outcomes only up to 12 months [34, 35]. The present analysis of the IN.PACT AV Access Study appears to be the first DCB RCT reporting long-term outcomes specific to cephalic arch stenosis with a primary patency rate of 40.9% through 3 years, a high rate for any endovascular treatment.

The three most common AVF types are the radiocephalic fistula (forearm), brachiocephalic, and brachiobasilic fistulas (upper arm), each with distinct stenosis sites [36]. Radiocephalic fistulas are preferred in patients requiring hemodialysis for longer than 12 months as they are associated with a lower risk of steal syndrome and better preservation of future access points [5, 37]. In the IN.PACT AV Access Study, radiocephalic AVFs had moderately better TLPP over brachiocephalic/brachiobasilic AVFs after 12 months for both DCB and PTA groups. The 36-month primary patency rate of radiocephalic AVF ranges from 28%−33% in the literature [38, 39], though primary patency definitions could be different. The DCB group in the present study showed a remarkable TLPP rate of 44.5% through 36 months that was significantly higher than PTA and favorable compared to rates reported in the literature. DCB also showed significantly higher TLPP through 36 months compared to PTA in the brachiocephalic/brachiobasilic AVF, suggesting that irrespective of the AVF type, DCB consistently outperforms PTA through 36 months.

We performed subset analyses by race and sex, finding that TLPP was higher with DCB than PTA across all races, with a greater, though not statistically significant, effect in Asian and Black participants compared to White participants. Similarly, DCB showed improved TLPP in both sexes, with a slightly greater, but not statistically significant, effect in men. A similar trend was also observed for the AcoArt Orchid DCB; however, data were reported only through 12 months [40]. Prior studies reported worse AVF outcomes for women compared to men [39, 41, 42] and for Black versus White patients [43]. However, insufficient data exist to determine optimal treatment strategies for these groups. Moreover, no previous studies have reported long-term DCB outcomes by race or sex for AVF stenosis; our study is the first to show long-term superior effectiveness of DCB versus PTA across races and sexes.

Previously, a meta-analysis of lower limb peripheral artery disease trials indicated increased long-term mortality with paclitaxel-coated devices compared to uncoated devices [44]. This concern was a key reason the FDA recommended extending the IN.PACT AV Access Study to 60 months to understand if there was a long-term mortality signal in this study. This study confirmed the long-term safety of DCB for AVFs, showing no increase in mortality over 60 months and comparable all-cause mortality rates to PTA. A plethora of real-world studies as well as prospective long-term studies with additional vital status ascertainment have not found any mortality signal with paclitaxel-coated devices [45–47]. The 60-month mortality rate of 41% in the DCB group of the present study is consistent with the published 60-month mortality rates of patients on hemodialysis, which are in the range of 37% to 69% [48–50].

Limitations: Because the analyses were conducted in subsets, the sample sizes were relatively small and may have been underpowered to detect the full treatment effect between groups; the exploratory nature of these analyses and the lack of adjustment for multiple comparisons may increase the risk of type I error. Therefore, findings should be interpreted with caution. Additionally, the trial's geographic distribution, limited to the United States, Japan, and New Zealand, as well as inclusion of only certain racial groups may limit the extrapolation of results to other regions with different healthcare systems or racial groups with different patient characteristics. This study used only one type of DCB, and these results may not be extrapolated to other DCBs.

Future research should focus on validating these findings in broader, real-world populations. Studies examining cost-effectiveness, patient-reported outcomes, and optimal surveillance strategies post-intervention would also be valuable. Furthermore, appropriate balloon sizing should be considered. A recent post-hoc analysis of IN.PACT AV Access RCT showed that balloons sized within 0.5 mm of the core laboratory adjudicated reference vessel diameter had the best outcomes throughout the trial [51]. While the use of intravascular ultrasound (IVUS) in the dialysis circuit has rarely been explored, IVUS may improve accurate vessel sizing and DCB outcomes in ESKD patients [52].

## Conclusions

The long-term outcomes from the IN.PACT AV Access Study advance the field by demonstrating that DCB offers a durable patency advantage over PTA and that this benefit extends across key anatomic and demographic subgroups. These results support the use of DCB in challenging lesions such as those that are restenotic, peri-anastomotic, and cephalic arch. The 5-year extension analysis demonstrated no mortality signal with the paclitaxel-coated IN.PACT AV DCB through 5 years.

## Supplementary Information

Supplementary Material 1: Table S1. Investigators list. Figure S1. Schematic diagram of AVF lesion treatment and definitions.

## Data Availability

To minimize the possibility of unintentionally sharing information that can be used to reidentify private information, anonymized data, and materials may be made available to qualified researchers trained in human subject confidentiality protocols after approval of a proposal and in accordance with sponsor policies and local regulatory guidelines.

## Abbreviations

AVF: Arteriovenous fistula
CEC: Clinical events committee
CI: Confidence interval
DCB: Drug-coated balloon
ESKD: End-stage kidney disease
FDA: Food and Drug Administration
HR: Hazard ratio
IVUS: Intravascular ultrasound
PTA: Percutaneous transluminal angioplasty
KDOQI: Kidney Disease Outcomes Quality Initiative
RCT: Randomized controlled trial
TLPP: Target lesion primary patency
