# Supplementary material for: IN.PACT AV Access randomized trial update: 3-year clinical results by anatomic and demographic subsets and 5-year extension mortality analysis
Source: CVIR Endovasc. 2026 Aug 19;9:105. doi: 10.1186/s42155-026-00746-z (PMC13490354; doi:10.1186/s42155-026-00746-z)
Supplement: Supplementary file 1 [file 42155_2026_746_MOESM1_ESM.docx]

**SUPPLEMENTARY APPENDIX**

**Table of Contents**

**Supplementary Table E1.** Investigators list

**Supplementary Figure E1.** Schematic diagram of AVF lesion treatment and definitions

**Supplementary Table E1. List of investigators**

| **Clinical Site** | **Location** | **Principal Investigator** | **Sub-Investigator** |
| --- | --- | --- | --- |
| **United States** |  |  |  |
| Cleveland Clinic | Cleveland, OH | Levester Kirksey | Federico Parod  David Hardy  Sean Lyden |
| Mayo Clinic | Rochester, MN | Sanjay Misra | Haraldur Bjarnason  Andrew Stockland  Newton Neider  Emily Bendel  Christopher  Reisenauer Melissa Neisen Erica Knavel |
| Sanford University of South Dakota (USD) Medical Center | Sioux Falls, SD | Angelo Santos Chad Laurich | Patrick Kelly |
| King's Daughters Medical Center | Ashland, KY | Omran Abul-Khoudoud | Alexander Hou Paul Lewis |
| The Mount Sinai Hospital | New York, NY | Adie Friedman | Michael Dudkiewicz Ronald Dreifuss |
| Holy Name Medical Center | Teaneck, NJ | John Rundback | Kevin Herman |
| University of Alabama at Birmingham (UAB) Hospital | Birmingham, AL | Husameddin El Khudari Ahmed Kamel Abdel Aal | Nathan Ertel Rachel Oser Andrew Gunn |
| University of Iowa Hospitals and Clinics | Iowa City, IA | Mel Sharafuddin | Sandeep Laroia Shiliang Sun Brendan O'Shea Brian Miller Timothy Kresowik William Sharp Shengfu Wang |
| Rush University Medical Center | Chicago, IL | Sreekumar Madassery |  |
| Sentara Vascular Specialists | Norfolk, VA | David Dexter | Samuel Steerman Animesh Rathore Richard DeMasi Gordon Stokes Scott McEnroe |
| University Surgical Associates | Chattanooga, TN | Charles Joels | Mark Fugate Michael Greer Larry Richard Sprouse Jeff Horn |
| Christie Clinic Vein and Vascular Center | Champaign, IL | Syed Hussain Nikolaos Karagiorgos | Jennifer Ash |
| Vascular Institute of Virginia | Woodbridge, VA | Sandeep Bagla Rachel Piechowiak |  |
| Richmond Vascular Center | North Chesterfield, VA | Jeffrey Hull |  |
| Dialysis Access Institute | Orangeburg, SC | Mark London John Ross | Jackson Ewart Jalal Hakmei Mohamed Sheta |
| North Carolina Nephrology | Raleigh, NC | Jeffrey Hoggard | Karn Gupta Sejan Patel |
| Florida Research Network | Gainesville, FL | Bret Wiechmann | Wesley Mann |
| Capital Nephrology Medical Group | Sacramento, CA | Naveen Atray | Rohit Kashyap Karthik Ramani |
| SKI Vascular Center | Tempe, AZ | Randy Cooper | Aslam Pervez Umar Waheed |
| Dallas Nephrology Associates | Plano, TX | Neghae Mawla | Steven Beathard |
| Coastal Vascular and Interventional | Pensacola, FL | Fernando Kafie | Huey McDaniel |
| **Japan** |  |  |  |
| Kansai Rosai Hospital | Amagasaki | Kotaro Suemitsu | Yuki Matsuoka Naomi Ota Kanako Oka Saho Kawanishi |
| Shonan Kamakura  General Hospital | Kamakura | Naoko Isogai Hidemitsu Ogino | Hidemitsu Ogino Katsunori Miyake Rai Shimoyama Naoko Isogai Jun Kawachi Takaaki Murata Nao Kum Yuto Igarashi Yuma Sunou Sumi Hidaka Kunihiro Ishioka |
| Kishiwada  Tokushukai Hospital | Kishiwada | Masahiko Fujihara | Yoshiaki Yokoi Akihiro Higashimori Nobuyuki Morioka Shinji Shiotani Keisuke Fukuda Tomofumi Tsukizawa Kensuke Kuwabara Yoshiki Matsuo Yuma Tanabe |
| Shizuoka General Hospital | Shizuoka | Masaaki Murakami | Noriko Mori Kiyoshi Mori Satoshi Tanaka Ken Matsuo Takao Okawa Shunsuke Okamura Yu Soma Yoshihiro Yamamoto Shota Kimura Yuki Ito Akira Sugawara Kenta Ito Ryo Yamada Yoko Matsuo Kakuya Hagiwara |
| Tokyo Women’s Hospital | Shinjuku-ku | Kazuhiro Iwadoh Ichiro Nakajima Shohei Fuchinoue | Sachiko Hirotani Kotaro Kai Yuichi Ogawa Katsuyuki Miki Takeshi Hachisuka Sayaka Morita Akira Kondo Kazuhiro Iwadoh |
| Saitama Medical Center Saitama Medical University | Kawagoe | Tomonari Ogawa | Hajime Hasegawa Toru Hida Taisuke Shimizu Nozomi Abe Tatsuro Sano Kunihiko Yasuda Tota Kiba Yoshimi Okada Koki Ogawa Hiroaki Hara Kento Hirose Yuichiro Kawai |
| **New Zealand** |  |  |  |
| Auckland City Hospital | Grafton | Andrew Holden | Brendan Buckley Brigid Connor Stephen Merrilees David Semple Andrew Hill |
| Capital & Coast District Health Board | Wellington | Janaka Kesara Wickremesekera (Kes Wicks) | Richard Evans Lupe Taumoepeau Anantha Narayanan Irina Baimatova |

| **Lesion Location** | | **Definitions** |
| --- | --- | --- |
| Peri-anastomotic | Arterial inflow | Treated segment is isolated to the arterial side |
|  | Anastomosis | Treated segment crosses or meets the arteriovenous anastomosis |
|  | Swing point | Treated segment includes the curved segment of mobilized vessel |
| Venous outflow | In cannulation zone | Treated segment is isolated to straight segment of vessel where cannulation is performed |
|  | Venous outflow | Treated segment is in basilic vein (non-mobilized) or distal to the cephalo-axillary junction |
| Cephalic arch | Cephalic arch | Treated segment includes curved segment of cephalic vein as the vein crosses between the pectoralis major and deltoid muscles |

**Supplementary Figure E1.** Schematic representation of AVF and key definitions. AVF, arteriovenous fistula.
